# Two-dimensional RMSD projections for reaction path visualization and validation^1^

**DOI:** 10.1016/j.mex.2026.103851

**Published:** 2026-03-04

**Authors:** Rohit Goswami

**Affiliations:** Institute IMX and Lab-COSMO, École polytechnique fédérale de Lausanne (EPFL), Station 12, CH-1015 Lausanne, Switzerland

**Keywords:** Reaction dynamics, Visualization, Machine learning potentials, Nudged Elastic Band, Saddle Points

## Abstract

Transition state or minimum energy path finding methods constitute a routine component of the computational chemistry toolkit. Standard analysis involves trajectories conventionally plotted in terms of the relative energy to the initial state against a cumulative displacement variable, or the image number. These dimensional reductions obscure structural rearrangements in high dimensions and may often be history dependent. This precludes the ability to compare optimization histories of different methods beyond the number of calculations, time taken, and final saddle geometry. We present a method mapping trajectories onto a two-dimensional projection defined by a permutation corrected root mean square deviation from the reactant and product configurations. Energy is represented as an interpolated color-mapped surface constructed from all optimization steps using a gradient-enhanced Gaussian Process with the inverse multiquadric kernel, whose posterior variance contours delineate data-supported regions from extrapolated ones. A rotated coordinate frame decomposes the RMSD plane into reaction progress and orthogonal distance. We show the utility of the framework on a cycloaddition reaction, where a machine-learned potential saddle and density functional theory reference lie on comparable energy contours despite geometric displacements, along with the ratification of the visualization for more complex reactions, a Grignard rearrangement, and a conrotatory bicyclobutane ring opening.

• **Dimensionality Reduction:** Maps optimization histories onto a 2D plane defined by distance-to-reactant and distance-to-product.

• **Landscape reconstruction:** Interpolates sparse optimization samples onto a continuous energy surface with data-driven smoothing to visualize basin topologies.

• **Validation:** Facilitates the direct projection of reference electronic structure calculations onto landscapes generated by machine-learned interatomic potentials.

## Graphical abstract

**Figure:**
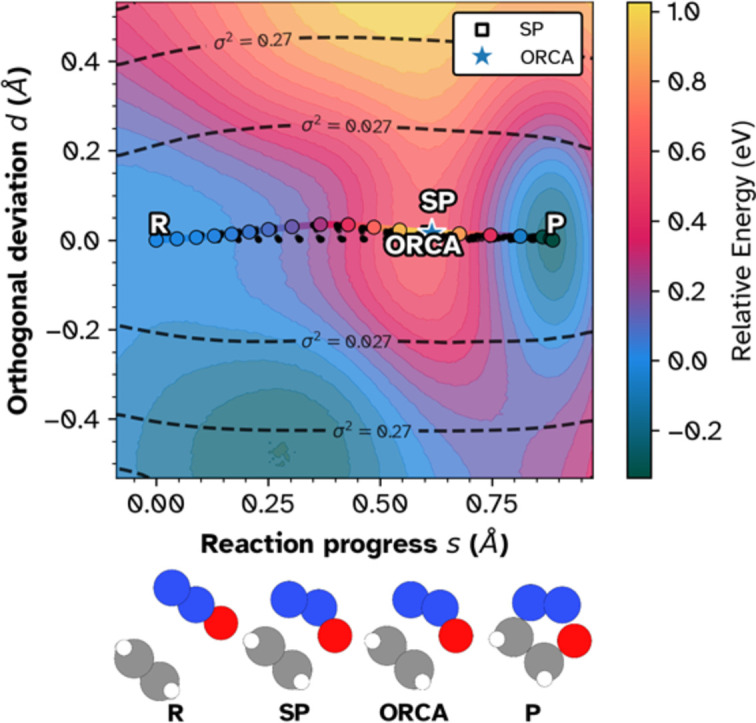

## Specifications table

**Table:** 

**Subject area**	Chemistry
**More specific subject area**	Chemical Physics / Reaction Dynamics
**Name of your method**	Intrinsic 2D RMSD Projection
**Name and reference of original method**	N/A
**Resource availability**	The rgpycrumbs package implements the frontend CLI: http://rgpycrumbs.rgoswami.me/ The chemparseplot package implements the backend surface visualization: https://github.com/HaoZeke/chemparseplot/ Github reproducer: https://github.com/HaoZeke/nebviz_repro Materials cloud archive: https://doi.org/10.24435/materialscloud:4b-vr

## Background

The first order saddle point is often the first approximation to understand the reaction kinetics of any system of interest. Starting from two known configurations, a series of configurations connected through fictitious springs, that is, the nudged elastic band method [1] is among the most common. The NEB may be coupled with the climbing image [2] and spring variations [3] with machine learned local accelerations [[4], [5], [6]] and the string family of methods [[7], [8], [9]]. Together, these constitute the current Pareto front for saddle searches.

Beyond convergence difficulties and the sensitivity to initial points, the resulting path histories are best studied through the “eye-ball norm”, wherein manual inspection of the final path, along with a normal mode analysis for the saddle point estimate are considered sufficient. In turn, this means the most common visual plots are one dimensional “profile” plots. These may show only the final optimized path, against the image number 2, which obscures any distance measure between the images. Alternatively, a well-known improvement to this involves the cubic Hermite interpolation [2] involving the forces relative to the “reaction coordinate,” defined by the piece-wise sum of the Euclidean distances between intermediate images. The reaction coordinate $s_{i}=\sum_{j=1}^{i}\left|R_{j}-R_{j-1}\right|$, depends entirely on the specific path geometry and optimization history. This scalar measure lacks a unique definition and collapses global geometric information onto a single, arbitrary axis. This dimensional reduction precludes rigorous comparison between saddles from differing algorithms or even identical algorithms with varying parameters. The one-dimensional projection frequently masks the distinction between numerical instability and physical relaxation into alternative basins. The resulting ambiguity in stationary point validation may cost days of calculations to clarify.

## Method details

**Fig. 1:**
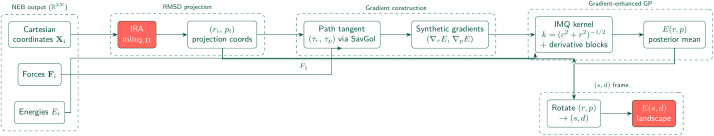
Overview of the 2D RMSD projection pipeline. NEB output in $R^{3N}$ is projected to intrinsic (r,p) coordinates via IRA-based permutation-invariant RMSD. Tangential forces are projected onto the 2D tangent to construct synthetic gradients. Energies and gradients jointly feed a gradient-enhanced GP with the IMQ kernel to produce the interpolated energy surface E(r,p). A final rigid rotation aligns the (r,p) frame to reaction progress s and orthogonal deviation d, yielding the E(s,d) landscape.

The complete methodology is summarized in Fig. 1. We describe the one-dimensional profiles first. Standard NEB implementations [10] provide discrete images **X**_i_ and energies E_i_. To reconstruct a physically consistent energy profile E(s) along the path, we use the available force information. We define the discrete reaction coordinate s_i_ as earlier, and instead of a simple linear or cubic interoplant, a Piecewise Cubic Hermite Interpolating Polynomial is used. This uses the tangent forces parallel to the path, $F_{∥,i}$ to constrain the derivative of the energy surface:

$$\frac{dE}{ds}{|}_{s_{i}}=-F_{∥,i}=-\left(F_{i}\cdot {\hat{\tau }}_{i}\right)$$

where ${\hat{\tau }}_{i}$ denotes the unit tangent vector along the path.

The 2D methodology consists of generating the unique coordinate set, mapping the optimization history, and interpolating through the subsequent energy landscape.

### Intrinsic projection coordinates

To map the high-dimensional optimization trajectory onto a consistent 2D subspace, we define the projection coordinates (r,p) as the distances from the reactant (R) and product (P) reference configurations. To ensure the metric remains robust across automated workflows where atom indexing may vary, we employ a permutation-invariant Root Mean Square Deviation (RMSD) with optimal permutation ∏ and rotation Q determined via the Iterative Rotations and Assignments (IRA) [11] algorithm. This procedure guarantees unique, invariant coordinates regardless of the initial atom indexing or frame orientation [6]. The discrete optimization steps provide a sparse sampling of this geometric subspace.

For a system with N atoms having positions $X\in R^{3\times N}$, we define the distance metric $d(X,X_{\text{ref}})$ as:

$$d\left(X,X_{\text{ref}}\right)=\underset{Q,}{\text{min}}\sqrt{\frac{1}{N}{∥X-QX_{\text{ref}}∥}_{F}^{2}}$$

Here, ${∥\cdot ∥}_{F}$ denotes the Frobenius norm. Q∈SO(3) represents the optimal rotation matrix, and ∏ represents the optimal permutation matrix. We solve for Q and ∏ simultaneously. This ensures that the resulting coordinates (r,p)=(d(X,R),d(X,P)) remain invariant to rigid body rotation, translation, and arbitrary atom index labeling.

### Reaction progress coordinates

The raw (r,p) plane contains an unphysical region where both RMSD distances are simultaneously small, which has no counterpart in configuration space for non-trivial rearrangements. We apply a rigid rotation that decomposes the RMSD plane into reaction progress s and orthogonal deviation d, analogous to the path collective variable decomposition [12]. Given the first and last path images with projected coordinates $\left(r_{0},p_{0}\right)$ and $\left(r_{N},p_{N}\right)$, we define the unit tangent $\left({\hat{s}}_{r},{\hat{s}}_{p}\right)$ along the path direction and the normal $\left({\hat{d}}_{r},{\hat{d}}_{p}\right)=\left(-{\hat{s}}_{p},{\hat{s}}_{r}\right)$. The rotated coordinates are then:

(4) $$s_{i}\;=\left(r_{i}-r_{0}\right){\hat{s}}_{r}+\left(p_{i}-p_{0}\right){\hat{s}}_{p}$$

(5) $$d_{i}\;=\left(r_{i}-r_{0}\right){\hat{d}}_{r}+\left(p_{i}-p_{0}\right){\hat{d}}_{p}$$

where s measures progress along the reaction path and d measures perpendicular deviation from it, both in Angstroms. The free energy literature [12] defines path progress and orthogonal distance via an exponential softmin over N reference frames, $s=\sum_{i}ie^{-\lambda R_{i}}/\sum_{i}e^{-\lambda R_{i}}$ and $z=-\left(1/\lambda \right)\text{ln}\sum_{i}e^{-\lambda R_{i}}$, with a smoothing parameter λ. That formulation targets enhanced sampling methods (metadynamics, umbrella sampling) where smooth, differentiable collective variables are required for biasing forces at finite temperature. For post-hoc visualization of a zero-temperature discrete path, the linear rotation suffices as it is parameter-free, exact at the endpoints, and directly invertible. Both decompositions separate progress along a path from deviation orthogonal to it. All figures in this work use this (s,d) frame.

Unlike principal component analysis (PCA) [13] or t-distributed stochastic neighbor embedding (t-SNE) [14] applied to covariance matrices [15,16], which require a priori selection of descriptors (e.g., bond lengths, angles, SOAP vectors [17]), RMSD-based projection operates directly on Cartesian coordinates without feature engineering. PCA-based methods popular in machine learning interatomic potential (MLIP) communities presuppose that the dominant variance directions align with chemically meaningful coordinates—an assumption that frequently fails for complex rearrangements involving concerted bond breaking and formation. Similarly, manifold learning techniques like t-SNE and UMAP [18] optimize for local neighborhood preservation but lack the absolute geometric reference frame necessary for quantitative cross-method comparison. Sketchmap [19] attempts to preserve both local and global distance structure, but requires on the order of $10^{4}$–$10^{5}$ samples to learn a reliable mapping, and even then does not guarantee metric preservation of absolute distances. A single NEB calculation produces $\approx 10^{3}$ geometries at most. Our endpoint-distance coordinates provide a universal, reaction-agnostic metric from exactly these $10^{3}$ points, with no training phase, no descriptor selection, and no presupposed chemical intuition about the reaction mechanism.

For systems with well-established collective variables (Ramachandran angles for peptides, donor-acceptor distances for proton transfer), such coordinates provide more direct physical insight [20]. However, the vast majority of transition state searches lack such convenient coordinates. Surface reactions, where an adatom hops into a subsurface site on a metallic slab, or catalytic processes involving concerted rearrangements of adsorbates, have no obvious low-dimensional collective variable. Constructing a two-dimensional free energy surface for such systems requires extensive sampling and a priori knowledge of the relevant degrees of freedom. In contrast, the RMSD-based projection requires only the Cartesian coordinates already produced by the calculation. Unlike free energy surface methods that mandate pre-specification of reaction coordinates, our approach visualizes arbitrary path based methods post hoc, requiring only the endpoints. This eliminates the circular dependency where one must already understand the mechanism to choose coordinates capable of revealing it.

Our method targets the complementary regime to global PES exploration techniques. It provides geometric validation for individual optimization trajectories ($\approx 10^{3}$ samples per NEB calculation) rather than exhaustive basin catalogs requiring $\approx 10^{6}-10^{9}$ samples. Most single NEB calculations involve only $\approx 10^{3}$ geometries concentrated along a one-dimensional path connecting a single reactant-product pair. This sampling is insufficient and inappropriate for techniques designed to map complete basin connectivity or construct disconnectivity graphs [21]. The framework instead asks whether this specific optimization found a physically reasonable barrier topology. For MLIP validation, where the goal is verifying that a machine-learned potential reproduces the correct transition pathway rather than exhaustively cataloging all possible pathways, this geometric diagnostic fills a gap that scalar comparisons cannot.

### Energy landscape projection

**Fig. 2:**
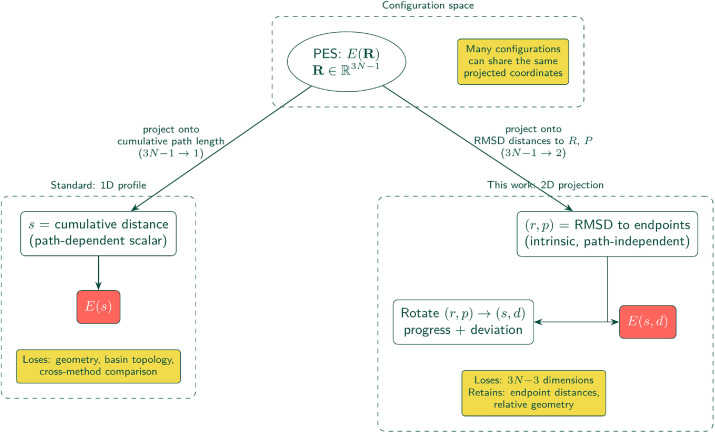
Dimensional reduction from the full 3N−1 configuration space to 1D (standard reaction coordinate s, left) versus 2D RMSD projection (this work, right). Both maps are many-to-one, and distinct Cartesian configurations can share the same projected coordinates. The 1D projection loses all geometric context. The 2D projection retains endpoint distances for cross-method comparison and landscape visualization, at the cost of 3N−3 unconstrained degrees of freedom.

The discrete optimization steps provide a sparse, unstructured sampling of the 2D (s,d) domain (Fig. 2). To visualize slices of the underlying potential energy landscape E(s,d), in a meaningful way to compare data from disparate calculations, we construct a continuous projection approximating the potential energy surface. Both the 1D and 2D projections are lossy, since multiple distinct configurations in $R^{3N}$ can map to the same projected coordinate. The 2D projection retains strictly more information than the 1D reaction coordinate, but it is not and cannot be the full PES.

While forces $F=-\nabla_{R}\left(E\right)$ are available from electronic structure calculations in 3N-dimensional Cartesian space, direct projection via the chain rule would require computing $\partial d_{\text{RMSD}}/\partial R$, a quantity complicated by the minimization over permutations and rotations implicit in the RMSD definition. Instead, we construct synthetic gradients in the 2D projection space by combining the path tangent vector with the available parallel force component.

First, we stabilize the tangent calculation via Savitzky-Golay smoothing of the RMSD coordinates themselves:

$${\tilde{r}}_{i},{\tilde{p}}_{i}=\text{SavGol}\left(r_{i},p_{i};\text{window}=5,\text{poly}=2\right)$$

From the smoothed coordinates, we compute the path tangent:

$$\tau_{r}=\frac{d\tilde{r}}{ds},\tau_{p}=\frac{d\tilde{p}}{ds}$$

where the tangent is normalized so | | *τ* | = 1. The projected gradient components are then:

$$\nabla_{r}E=-F_{∥}\cdot \tau_{r},\nabla_{p}E=-F_{∥}\cdot \tau_{p}$$

where $F_{∥}$ is the force component parallel to the path (column 3 in an eOn .dat file). This construction ensures gradient information respects the local path geometry while remaining tractable to compute from standard NEB output.

Only the tangential force component $F_{∥}$ enters this construction. The full Cartesian force $F_{i}\in R^{3N}$ contains orthogonal components that encode local curvature of the true PES. Projecting these into the (r,p) subspace would require the Jacobian $\partial d_{\text{RMSD}}/\partial R$, which is not analytically available. The RMSD involves a joint minimization over rotations Q∈SO(3) and permutations ∏, and the resulting map from $R^{3N}$ to $R^{2}$ is piecewise smooth at best, nondifferentiable boundaries wherever the optimal permutation assignment changes. This is not a software limitation but a mathematical property of the permutation-invariant RMSD itself. The tangential projection is therefore the consistent choice without additional approximations.

The omission of orthogonal force components means the interpolated surface cannot reproduce the curvature perpendicular to the path. In the full 3N-dimensional PES, MEP images are by definition minima in the hyperplane orthogonal to the path; the 2D projection has no mechanism to enforce or verify this property. The tangential projection nonetheless carries non-trivial 2D information because the tangent direction $\left(\tau_{r},\tau_{p}\right)$ varies along the path. Force information is distributed across both projected coordinates in a way that scalar reaction coordinate plots cannot access.

The interpolated surface is a projected slice of the full 3N-dimensional PES. Many distinct Cartesian configurations map to the same (r,p) point. The interpolation represents the conditional expectation of the energy given the RMSD coordinates, marginalizing over the unconstrained 3N−3 degrees of freedom. Features of the projected surface, such as apparent alternative pathways in the color map, do not necessarily correspond to physical pathways in the full configuration space. Similarly, the property that MEP images are minima in the orthogonal hyperplane holds in the full-dimensional PES but has no analogue in a two-dimensional lossy projection. The interpolation is reliable near the sampled data and degrades with distance, as with any regression model.

We denote the 2D projection coordinates compactly as $x_{i}=\left(r_{i},p_{i}\right)=\left(d_{\text{RMSD}}\left(S_{i},R\right),\;d_{\text{RMSD}}\left(S_{i},P\right)\right)$. Together with the projected gradients $\left(\nabla_{r}E,\nabla_{p}E\right)$ from above, these form the inputs to a Gaussian process regression with derivative observations [[22], [23], [24]]. We construct the energy surface using the Inverse Multiquadric (IMQ) kernel:

$$k\left(x,x{}^{\prime }\right)={\left(c^{2}+r^{2}\right)}^{-1/2},r^{2}=∥x-x{}^{\prime }{∥}^{2}$$

where c is a scale parameter. The function $g\left(t\right)={\left(c^{2}+t\right)}^{-1/2}$ is completely monotone on [0,∞): ${\left(-1\right)}^{n}g^{\left(n\right)}\left(t\right)\geq 0$ for all n≥0. By Schoenberg’s theorem [25], any kernel of the form $k=g(∥x-x{}^{\prime }{∥}^{2})$ with g completely monotone is strictly positive definite on $R^{d}$ for all d [26]. This guarantees non-singular interpolation matrices regardless of point configuration.

We augment the standard energy-only covariance matrix with derivative observations. For each sampled point $\left(x_{i},y_{i}\right)=\left(d_{\text{RMSD}}\left(S_{i},R\right),d_{\text{RMSD}}\left(S_{i},P\right)\right)$, we include the projected gradients $\left(\nabla_{x}E,\nabla_{y}E\right)$ computed as described above. This constructs an augmented observation vector $y_{\text{full}}=\left[E_{1},\nabla_{x}E_{1},\nabla_{y}E_{1},E_{2},\nabla_{x}E_{2},\nabla_{y}E_{2},\ldots \right]$ with corresponding gradient-augmented kernel blocks :

$$K_{\text{full}}=\left[\begin{array}{cc} k\left(x_{i},x_{j}\right) & \nabla_{x{}^{\prime }}k\left(x_{i},x_{j}\right)\\ \nabla_{x}k\left(x_{i},x_{j}\right) & \nabla_{x}\nabla_{x{}^{\prime }}k\left(x_{i},x_{j}\right) \end{array}\right]$$

where the derivative covariances are computed via automatic differentiation using the JAX library, so extending to other kernels requires only changing the base kernel function. For the IMQ these are:

$$\begin{array}{cc} \frac{\partial k}{\partial x_{i}} & =-\left(x_{i}-x_{i}{}^{\prime }\right)\;{\left(c^{2}+r^{2}\right)}^{-3/2}\\ \frac{\partial^{2}k}{\partial x_{i}\partial x_{j}{}^{\prime }} & =\delta_{ij}{\left(c^{2}+r^{2}\right)}^{-3/2}-3\left(x_{i}-x_{i}{}^{\prime }\right)\left(x_{j}-x_{j}{}^{\prime }\right){\left(c^{2}+r^{2}\right)}^{-5/2} \end{array}$$

which assemble into the augmented kernel matrix [27].

As r→∞, the IMQ kernel decays as $r^{-1}$, its first derivatives as $r^{-2}$, and second derivatives as $r^{-3}$. This polynomial decay contrasts with the exponential decay of the Matérn and squared exponential families. Heavy tails allow the kernel to capture long-range basin structure from sparse path data, and the augmented kernel matrix remains better conditioned than exponentially decaying counterparts. Including derivative observations effectively triples the information content per sampled geometry without additional energy evaluations.

The hyperparameter optimization follows the subset optimization [28] concept from the OT-GPD, here, the points from the final path are used to calculate the length and noise scales, which are subsequently applied while fitting the entire surface. This subsampled approach reduces computational cost from $O(N^{3})$ to $O(n^{3})$ where n≪N (typically n≈20 vs N≈500−2000), while simultaneously improving robustness by isolating hyperparameter learning from transient optimization dynamics.

The GP posterior naturally provides a pointwise variance estimate. We overlay variance contours on the energy surface to delineate data-supported regions from extrapolated ones. Near the sampled path, the posterior variance is small; it grows with distance from the data and serves as a built-in reliability indicator for the interpolation.

For systems producing more than approximately $10^{3}$ gradient-augmented observations (e.g., periodic slab calculations with many NEB steps), the $O(N^{3})$ cost of the full GP becomes prohibitive. We use a Nystrom low-rank approximation [29] adapted to the gradient-enhanced setting. Given M≪N inducing points ${\left\{x_{m}\right\}}_{m=1}^{M}$ selected from the training set, the augmented kernel matrix $K_{NN}$ (of size N(D+1)×N(D+1), where D=2 is the input dimension) is approximated as

(12) $${\tilde{K}}_{NN}\approx K_{NM}K_{MM}^{-1}K_{MN}$$

where $K_{NM}$ and $K_{MM}$ are the cross and inducing kernel matrices, each containing the value, gradient, and Hessian blocks of the IMQ kernel. The predictive mean becomes $\overset{ˆ}{f}\left(x_{*}\right)=k_{*M}\alpha_{M}$ with weights obtained from

(13) $$\alpha_{M}=L^{-⊤}\left(S^{-1}Vy_{\text{full}\;}\right),\;S=VV^{⊤}+\sigma^{2}I_{M\left(D+1\right)}$$

where L is the Cholesky factor of $K_{MM}+\sigma^{2}I$ and $V=L^{-1}K_{MN}$. Inducing points are selected from the last converged NEB path(s), which concentrate observations near the final MEP geometry. This reduces the dominant cost from $O(N^{3})$ to $O(NM^{2})$ (typically =300,N>5000) and makes the method applicable to crystalline systems.

The resulting interpolated surface enables direct overlay of reference structures (e.g., DFT-optimized saddle points) onto MLIP-generated landscapes. One can then assess whether a potential captures qualitatively correct barrier topology even when geometric displacements occur. This is impossible with 1D energy profiles, where the reaction coordinate axis itself depends on path geometry.

## Method validation

**Fig. 3:**
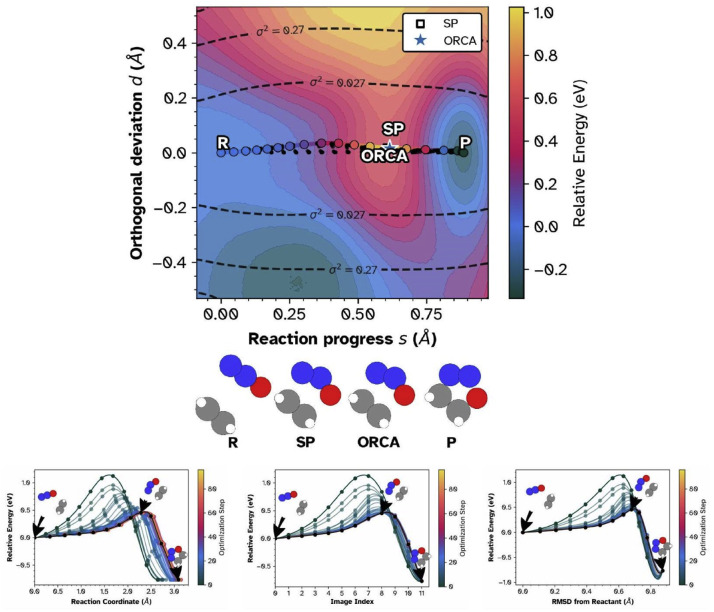
NEB optimization of ethylene + N2O cycloaddition using PET-OMAT potential. **Top:** 2D projection in (s,d) coordinates (reaction progress vs. orthogonal distance) showing interpolated energy landscape (color), sampled structures (black dots), converged path (open circles), and posterior variance contours (dashed lines). The white star indicates ORCA B3LYP-D3 saddle. **Bottom left:** Energy vs. reaction coordinate. **Bottom center:** Energy vs. image index. **Bottom right:** Energy vs. RMSD from reactant. In all panels, colored curves show optimization progression (dark→light = early→late); final path in black.

We empirically demonstrate the utility of the framework, first contrasting against 1D profiles for the 1,3-dipolar cycloaddition of ethylene and N2O forming 4,5-dihydro-1,2,3-oxadiazole, a well-studied benchmark reaction for NEB method development [3,5,9,[30], [31], [32]]. Fig. 3 compares conventional one-dimensional representations with the two-dimensional projection for energy-weighted NEB optimization in eOn [33]^3^ using the PET-OMAT machine-learned potential [34,35] using Metatomic [30]. The model has been trained on PBE reference data from the Open Materials dataset [36], with full construction details in the associated publication. For the cycloaddition, we contrast this with a saddle optimized from a different NEB calculation using the B3LYP functional in ORCA [3] projected onto the interpolated landscape.

The conventional profiles (Fig. 3, bottom) show optimization from an initial barrier of approximately 1.1 eV to a final value near 0.4 eV over 120 steps, with the product lying approximately 0.8 electron-volt below the reactant. The final path appears smooth and well converged. These representations provide no information about sampling quality or the broader landscape topology.

The two-dimensional projection (Fig. 3, top) provides. Sampled structures (black dots) cluster tightly along the converged path, confirming robust single-pathway convergence. The interpolated energy surface displays a barrier region (mauve/orange, approximately 0.4 eV) separating the reactant basin from the deeper product basin (blue, right). The projection reveals that the ORCA saddle configuration overlaps with the estimate from the MLIP. The posterior variance contours (dashed lines) confirm that the interpolation is tightly constrained near the path and visibly degrades in the periphery, serving as a built-in reliability indicator for the surface estimate.

**Fig. 4:**
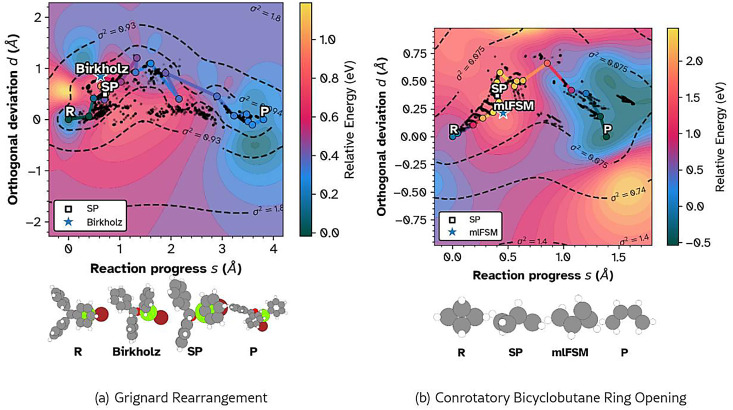
Two-dimensional (s,d) projections of complex rearrangement pathways with posterior variance contours (dashed lines). Both surfaces use the Nyström low-rank GP approximation; darker points denote the inducing subset (final converged NEB steps) while lighter points show the remaining optimization history retained for context. (a) The Grignard rearrangement of (Z)-2-phenyl-2-((trimethylsilyl)oxy)hex‑4-enenitrile exhibits a distinct “turning” mechanism. The projection reveals optimization instability (scattered transparent black dots) near the reactant basin that 1D profiles would obscure. The star indicates the reference saddle from Birkholz et al. (b) The conrotatory ring opening of bicyclobutane shows a sharp geometric “kink” post-transition state. The projection confirms the machine-learned potential path (circles) faithfully traverses the saddle region identified by the Free String Method (star).

To assess robustness on non-trivial topologies, we analyze the Grignard rearrangement of (Z)-2-phenyl-2-((trimethylsilyl)oxy)hex‑4-enenitrile [32] and the conrotatory ring opening of bicyclobutane (Fig. 4) [9]. Un-like the linear character of the cycloaddition, these reactions proceed through significant geometric turns. The Grignard pathway (Fig. 4a) exhibits a curved trajectory where the path tangent rotates nearly 90 degrees in the (s,d) plane. The 2D projection captures the optimization noise (scattered lighter points) near the reactant basin, a diagnostic feature lost in scalar reaction coordinate plots, while showing without explicit structural inspection that the reference and computed saddles lie on the same energy contour. conrotatory bicyclobutane ring opening (Fig. 4b) displays a sharp geometric “elbow” immediately following the transition state. The interpolated surface correctly places the reference Free String Method (mlFSM) saddle [9] within the barrier region of the MLIP. This confirms that the NEB trajectory found the physically relevant saddle despite the complex topology, a validation that remains ambiguous when observing only the image index or scalar reaction coordinate, which in practice would involve normal mode analysis, barrier heights, RMSD, and visual inspection.

The 2D projection provides information beyond standard scalar comparisons. Computing energy differences and RMSD values between two saddle points tells one *that* they differ, but not *how* they differ relative to the reaction landscape. A 1D energy profile, by construction, collapses all geometric information onto the cumulative displacement axis. It cannot distinguish whether two saddle estimates from different methods lie on the same energy contour, sit in different basins, or whether the optimization explored alternative pathways before converging. The 2D projection preserves these geometric relationships. One can immediately see, for example, that the ORCA and MLIP saddles in Fig. 3 occupy the same barrier region despite different path histories, or that the optimizer noise in the Grignard case (Fig. 4a) is confined to a specific geometric region rather than distributed along the path. These are qualitative assessments, analogous to plotting a trajectory on a map rather than reporting only the total distance traveled.

The framework also provides a unified coordinate system for validating potential energy surfaces against higher-level theory. If these disagree, the relative differences between the saddles are apparent. The projections reveal whether resulting structures sit within comparable energy contours. Determining whether the energy surface captures the qualitative barrier topology, even when the precise saddle geometry differs due to functional sensitivity is impossible with conventional one-dimensional profiles.

For complete reproduction, the landscape figures are generated by a single command-line invocation. As an example, the Grignard projection (Fig. 4a) is produced by:



The –project-path flag (on by default) rotates the raw (r,p) RMSD coordinates into the (s,d) frame described above, –surface-type grad_imq_ny selects the Nystrom-accelerated gradient-enhanced IMQ kernel, –ira-kmax sets the adjacency cutoff distance for IRA graph-based structure matching, –plot-structures crit_points renders the reactant, product, saddle estimate, and highest-energy image, and –additional-con overlays a reference saddle from an external file. Cosmetic options (figure size, DPI, font size, rotation) are omitted for brevity. The materials archive includes exact commands and outputs at the time of submission.

### Limitations

The (r,p) projection is a lossy map from $R^{3N}$ to $R^{2}$. Multiple distinct Cartesian configurations can share the same RMSD coordinates, so the interpolated energy surface does not reconstruct the true PES but rather a projected slice of it. Apparent features far from the sampled data, for instance, alternative low-energy pathways visible in the color map, may not correspond to physical pathways in the full configuration space. The interpolation should be read as a qualitative guide near the data, not as a quantitative surrogate for the potential. The posterior variance contours overlaid on the figures directly visualize this degradation so that users can distinguish data-supported features from extrapolation artifacts.

As discussed in the methodology, only the tangential force component enters the gradient projection. The method does not recover curvature information orthogonal to the path, and the MEP orthogonality property cannot be enforced in the 2D projection. Any extension to orthogonal force projection would require differentiable structure matching. For molecular systems where the permutation group is large, this remains an open problem. For crystalline systems, where space group symmetry constrains the permutation set, tractable approximations may be feasible and are a natural direction for future work.

The choice of IMQ kernel, while theoretically motivated by its positive definiteness and polynomial tail properties, is one of several valid options. The software provides squared exponential, Matern 5/2, and thin plate spline alternatives. Different kernels may be better suited to different reaction topologies, and no single kernel is universally optimal.

Finally, the method is designed for post-hoc visualization of existing calculations. It does not accelerate the NEB computation itself, nor does it replace quantitative analysis (normal mode analysis, barrier heights, vibrational frequencies) required for kinetic rate calculations.

## Conclusions

We presented a coordinate-free visualization method for analyzing reaction path optimization trajectories. By projecting high-dimensional pathways onto a surface defined by permutation-invariant RMSD coordinates, the method reveals geometric and energetic features obscured by standard one-dimensional profiles.

In current practice, comparing NEB results across methods or potentials requires either pointwise structural inspection (impractical beyond a handful of images) or scalar summaries (barrier height, RMSD to reference) that discard geometric context. The 2D projection occupies the space between these extremes. A single plot preserves the geometric relationship between all sampled structures, the converged path, and any reference points, while remaining interpretable at a glance. The benchmarks show that this representation distinguishes numerical artifacts from physical topology and reveals whether different potentials produce qualitatively similar barrier regions, even when the precise saddle geometries differ. The rotated (s,d) frame separates reaction progress from path deviation and gives a more physical coordinate system than raw RMSD distances. The posterior variance contours supply built-in reliability assessment for the interpolated surface. A Nystrom low-rank approximation extends the method to crystalline systems with large numbers of observations.

While we focus on double-ended methods here, the projection operates independently of the path generation algorithm. One can in principle project histories from single-ended saddle search methods, molecular dynamics, or metadynamics onto these intrinsic axes post-hoc to diagnose path quality and hysteresis with a destination basin. Additionally, the relative energy surfaces from multiple machine learning models could also be probed within such a framework.

## Ethics statements

Not applicable.

## Declaration of competing interest

Not applicable.

## Data Availability

Reproduction materials at the Materials Cloud Archive https://doi.org/10.24435/materialscloud:4b-vr.
